# The atmospheric oxidizing capacity and the methane budget

**DOI:** 10.1093/nsr/nwaf347

**Published:** 2025-08-21

**Authors:** Guy P Brasseur, Benjamin Gaubert

**Affiliations:** NSF National Center for Atmospheric Research (NSF NCAR), USA and Max Planck Institute for Meteorology, Germany; NSF National Center for Atmospheric Research (NSF NCAR), USA

The increase in atmospheric methane (CH₄) is of concern because this greenhouse gas has a high global warming potential. After an 8-year pause (2000–2007) in the atmospheric methane growth trend and a resumption that has accelerated since 2007, a surge in the global atmospheric methane burden was observed in 2020, 2021 and 2022, with an unprecedently high atmospheric growth rate. Analysis of the methane isotopic content [1], as well as satellite observations (e.g. [2]) led to the attribution of the resumed methane growth (post 2007) to microbial emissions, which are predominantly from tropical wetlands, with contributions from waste and agricultural activity.

Earth system models with interactive chemistry and inversion method estimations from methyl-chloroform observations currently disagree on the temporal trends in the hydroxyl radical (OH), the chemical species responsible for 90% of atmospheric methane oxidation [3]. However, there is a growing consensus that the global OH inter-annual variability is <4% [4].

The OH radical is primarily formed through ozone (O_3_) photolysis and ‘recycled’ with nitrogen oxides’ (NOx) photocatalytic cycles, while it is destroyed by CH_4_, carbon monoxide (CO) and many volatile organic species. This has led to an increased interest in characterizing OH at a shorter temporal scale from satellite observational proxies of OH sources and sinks of such species [5]. The COVID-19 pandemic provides an excellent case study to apply such techniques with months-long hemispheric scale anomalies in O_3_ levels and NO_x_ emissions, as quantified by assimilated satellite observations [6], ozone profile observations and model simulations (e.g. [7,8]).

The paper by Chen *et al.* [9] specifically addresses the question of the 2020 methane anomaly. From a detailed investigation based on data analysis and modeling, the Westlake University researchers conclude that the global mean OH concentration was ∼4% lower in 2020 compared to 2018–2019 (2.4% in the Northern Hemisphere and 5.7% in the Southern Hemisphere). The authors inferred the tropospheric concentration of OH for each season and for six latitude bands using an inversion algorithm that assimilates satellite (MOPITT) observations of the CO column.

The explanation proposed by Chen *et al.* [9] for this 2020 anomaly refers to forcing mechanisms that are different in the two hemispheres. South of the equator, the reduction in OH is attributed to an anomalous increase in the CO concentration resulting from the intense Australian wildfires, thereby facilitating a rapid methane growth. North of the equator, the OH decrease is attributed to the large decrease in NO_x_ emissions observed during the COVID-19 pandemic, and hence a reduced recycling of OH. From an inverse analysis of methane satellite data, the authors determined that the observed 27 Tg_CH4_ a^−^^1^ increase in the methane burden, relative to years 2018–2019, results from a 17 Tg_CH4_ a^−^^1^ increase in surface emissions, and an 18 Tg_CH4_ a^−^^1^ reduction in the atmospheric loss by OH, which is partly compensated by an 8 Tg_CH4_ a^−^^1^ increase in the loss, due to enhancement of the methane concentration.

The study by Chen *et al.* [9] highlights the strong link that exists between the variations and trends in the atmospheric abundance of some climate-relevant atmospheric constituents, such as methane, and air pollutants such as CO, NO_x_ and ozone. This highlights the need to include the influence of chemical processes in climate simulations and projections. An important extension of the study performed by Chen *et al.* [9] would be to assess how the exceptional situation of 2020 changed the relative contribution of different chemical processes to the OH production and destruction rates. Improved quantification of the methane budget can only be achieved by reducing uncertainty in the OH budget, through analyzing processes with chemistry-climate models and innovative use of chemical observational datasets.
